# Repurposing Ponatinib Hydrochloride as an Antibacterial and Antibiofilm Agent Against Multidrug-Resistant *Staphylococcus aureus*

**DOI:** 10.3390/microorganisms14092066

**Published:** 2026-09-16

**Authors:** Xinhua Wang, Na Kong

**Affiliations:** College of Pharmacy, Heze University, Heze 274015, China

**Keywords:** drug repurposing, ponatinib hydrochloride, antibacterial, antibiofilm, MRSA

## Abstract

With the continuous escalation of antimicrobial resistance, drug repurposing based on approved drugs has emerged as a promising strategy for the development of novel anti-infective agents. This study aimed to evaluate the antibacterial effect of the FDA-approved drug Ponatinib hydrochloride against multidrug-resistant *S. aureus*. Broth microdilution assay showed that ponatinib hydrochloride exhibited potent activity against Gram-positive bacteria, while the MICs against Gram-negative bacteria were all >100 μM. In addition, the MICs against 15 clinical multidrug-resistant *S. aureus* isolates ranged from 6.25 to 25 μM. Time-kill assay further revealed a pronounced concentration- and time-dependent bactericidal effect, with bacterial viability reduced to nearly the detection limit within 24 h at 4 × MIC. Anti-biofilm assays showed that ponatinib hydrochloride significantly inhibited *S. aureus* biofilm formation and disrupted mature biofilms. Moreover, serial passaging for 30 days did not induce an obvious increase in resistance. Mechanistic investigations demonstrated that ponatinib hydrochloride increased membrane permeability, induced intracellular reactive oxygen species (ROS) accumulation, and markedly reduced ATP levels. Finally, in a mouse infection model, ponatinib hydrochloride significantly improved the survival of infected mice, with a survival rate of 70% observed in the 15 mg/kg treatment group, while also reducing bacterial burdens and pro-inflammatory cytokine levels in tissues. Collectively, ponatinib hydrochloride exhibits potent antibacterial activity against multidrug-resistant *S. aureus* both in vitro and in vivo and represents a promising candidate for anti-MRSA therapy.

## 1. Introduction

*S. aureus* is an important zoonotic opportunistic pathogen that can cause a wide range of diseases, including skin and soft tissue infections, pneumonia, bacteremia, sepsis, and infective endocarditis, and in severe cases may lead to multiple organ failure and death [1]. Owing to its strong environmental adaptability and virulence factor expression, *S. aureus* has become one of the major pathogens responsible for both hospital-acquired and community-acquired infections worldwide [2]. In recent years, the widespread and irrational use of antibiotics has led to the increasing prevalence of drug-resistant *S. aureus*, among which methicillin-resistant *S. aureus* (MRSA) has emerged as one of the most concerning “superbugs” in global public health [3]. In addition, MRSA can spread in both hospital and community settings, and its epidemiology has gradually shifted from traditional hospital-acquired MRSA to community-acquired MRSA, further complicating prevention and control efforts [4]. Currently, the primary agents used for the treatment of MRSA infections include vancomycin, linezolid, and daptomycin [5]; however, prolonged use of these antibiotics may lead to the emergence of new resistance and adverse side effects [6]. Moreover, MRSA possesses a strong ability to form biofilms, which further enhances its tolerance to host immune responses and antimicrobial agents [7]. Therefore, the development of novel antibacterial agents or alternative therapeutic strategies has become an important priority in current anti-infective research.

The development of antibacterial agents has significantly lagged behind the rapid evolution of bacterial resistance, and antimicrobial resistance (AMR) has become one of the most severe challenges in global public health [8]. According to the World Health Organization (WHO) antibacterial pipeline report, only 12 new antibiotics have been approved since 2017, of which 10 still belong to existing antibiotic classes for which bacteria have already developed corresponding resistance mechanisms [9]. Truly novel antibiotics with innovative mechanisms of action remain extremely limited. This situation indicates that the traditional antibiotic development model is no longer sufficient to effectively address the escalating resistance crisis. Currently, most clinically used antibiotics exert bactericidal effects by inhibiting cell wall synthesis, protein synthesis, or DNA replication [10]. However, these direct bactericidal strategies impose strong selective pressure on bacteria, thereby accelerating the emergence and dissemination of resistant strains [11]. In addition, conventional antibiotics exhibit considerable limitations in eradicating biofilms. Studies have shown that bacteria within biofilms often display high antimicrobial tolerance due to reduced metabolic activity, limited drug penetration, and enhanced exchange of resistance genes [12]. Under these circumstances, drug repurposing has gradually emerged as an important strategy for anti-infective drug development because of its shorter development cycle, lower cost, and well-established safety profiles [13]. Approved drugs have generally already undergone pharmacokinetic, safety, and toxicological evaluations, which can substantially shorten clinical translation time and reduce the risk of drug development failure [14]. In recent years, several non-antibiotic drugs have been reported to possess potential antibacterial, antibiofilm, or antibiotic-sensitizing activities [15,16,17], providing new perspectives for the treatment of drug-resistant bacterial infections.

Ponatinib hydrochloride is a third-generation multi-target tyrosine kinase inhibitor (TKI) and was approved by the United States Food and Drug Administration (FDA) in 2012 [18]. It is primarily used for the treatment of chronic myeloid leukemia (CML) and Philadelphia chromosome-positive acute lymphoblastic leukemia (Ph+ ALL) [19]. Previous studies have reported that Ponatinib can function as an effective inhibitor of the cell division protein FtsZ in *Fusobacterium nucleatum*, thereby exerting antibacterial activity [20]. These findings provide a new theoretical basis for the application of Ponatinib in the antibacterial field. However, the antibacterial activity and underlying mechanisms of Ponatinib against *S. aureus*, particularly MRSA, remain largely unexplored. Considering the strong drug resistance and biofilm-forming ability of MRSA, investigating the anti-MRSA potential of Ponatinib is of considerable significance for expanding its value in drug repurposing. Therefore, this study aimed to evaluate the antibacterial and antibiofilm activities of ponatinib hydrochloride against MRSA and further explore its potential mechanisms of action, with the goal of providing a novel candidate agent and theoretical basis for the treatment of drug-resistant *S. aureus* infections.

## 2. Results

### 2.1. Ponatinib Hydrochloride Exhibits Potent In Vitro Antibacterial Activity Against Multidrug-Resistant S. aureus

**Figure 1:**
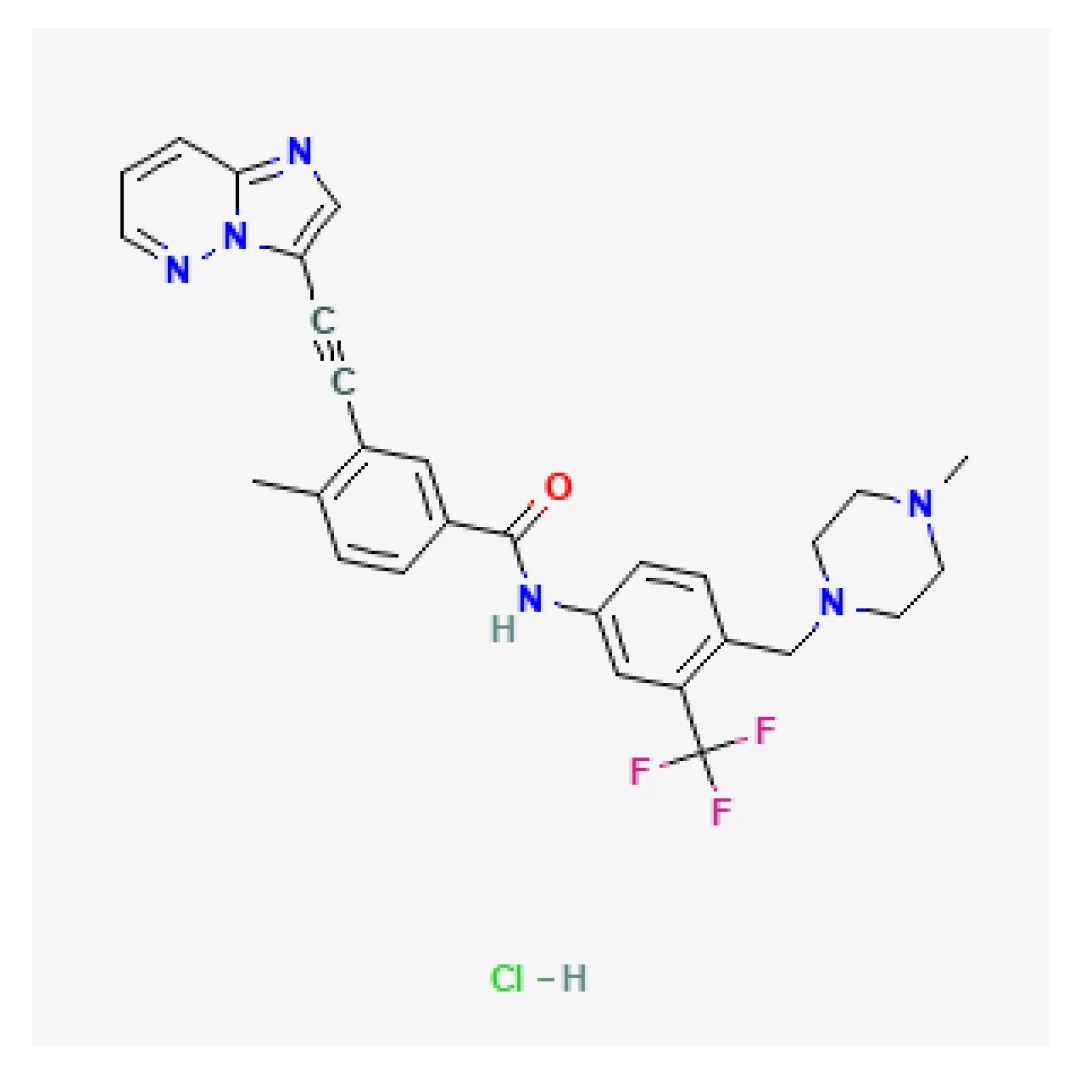
The 2D structure of ponatinib hydrochloride. The structure is derived from the NCBI database (CID: 46908927) and the molecular weight is 569.000 g/mol.

The structure of ponatinib hydrochloride is shown in Figure 1. As shown in Table 1, ponatinib hydrochloride exhibited favorable antibacterial activity against Gram-positive bacteria. The MICs against MSSA ATCC 29213 and MRSA ATCC 43300 were 6.25 μM and 12.5 μM, respectively, while the MIC against *S. pneumoniae* ATCC 49619 was 12.5 μM. In contrast, the MICs against the Gram-negative bacteria *E. coli* ATCC 25922 and *P. aeruginosa* ATCC 9027 were both >100 μM, indicating that the antibacterial activity of ponatinib hydrochloride was mainly directed against Gram-positive bacteria. Furthermore, the susceptibility of 16 clinical multidrug-resistant *S. aureus* isolates to ponatinib hydrochloride was evaluated. These isolates exhibited varying multidrug-resistant phenotypes. Nevertheless, ponatinib hydrochloride maintained strong antibacterial activity against these clinical MRSA isolates, with MIC values ranging from 6.25 to 25 μM. Most isolates exhibited MICs between 6.25 and 12.5 μM, whereas only a few strains showed MICs of 25 μM. Collectively, these results demonstrate that ponatinib hydrochloride possesses potent in vitro antibacterial activity against both standard strains and clinical multidrug-resistant *S. aureus* isolates, and its antibacterial efficacy remains stable under multidrug-resistant backgrounds, highlighting its potential as a candidate agent for the treatment of multidrug-resistant *S. aureus* infections.

### 2.2. Ponatinib Hydrochloride Shows Concentration-Dependent Bactericidal Activity Against S. aureus

**Figure 2:**
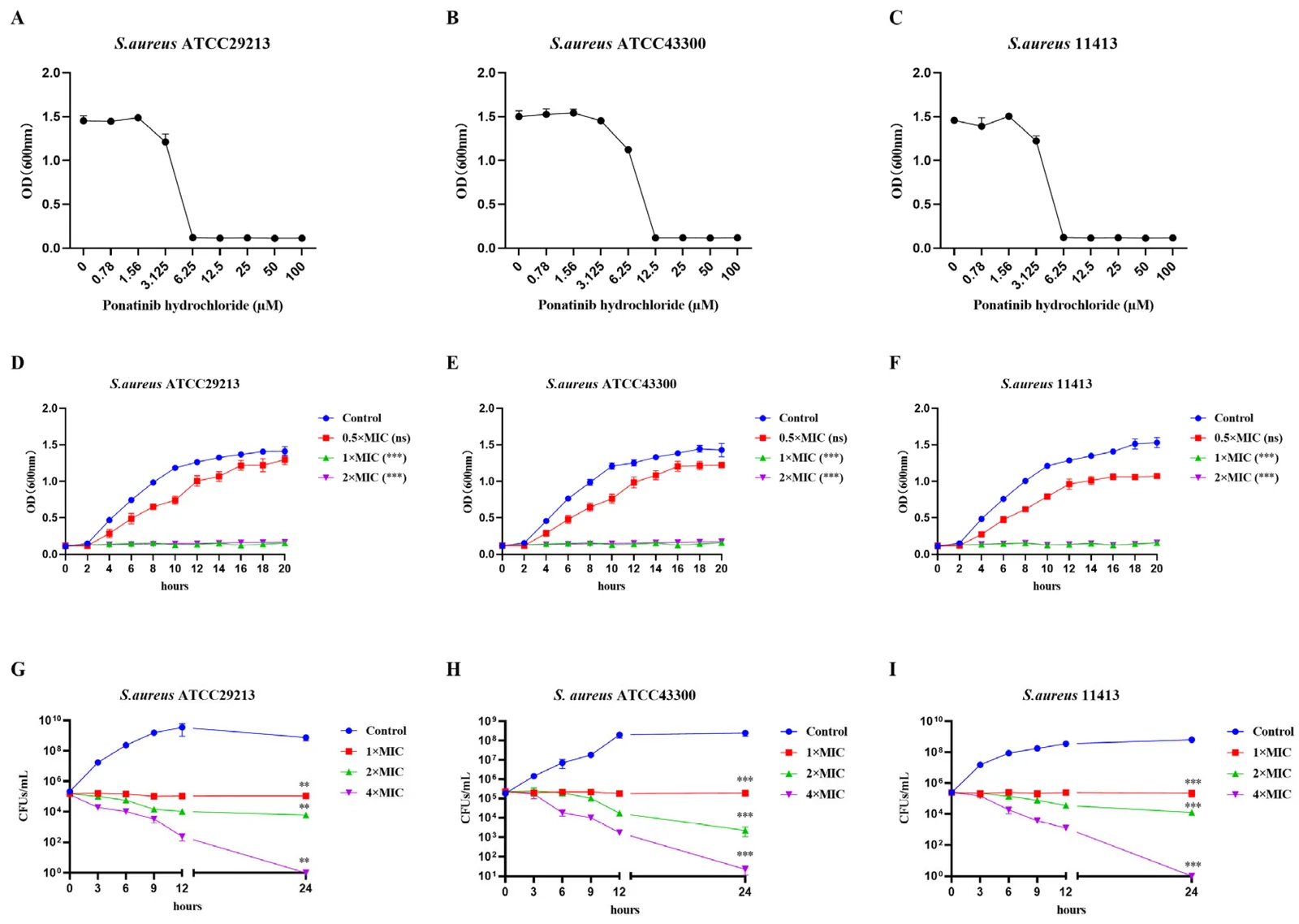
Antibacterial activity of ponatinib hydrochloride against *S. aureus*. (**A**–**C**) Changes in OD600 values of *S. aureus* ATCC 29213, MRSA ATCC 43300, and the clinical multidrug-resistant *S. aureus* isolate 11413 following treatment with different concentrations of ponatinib hydrochloride. (**D**–**F**) Effects of ponatinib hydrochloride at 0.5 × MIC, 1 × MIC, and 2 × MIC on the growth curves of the three *S. aureus* strains. (**G**–**I**) Time-kill curves of the three *S. aureus* strains treated with ponatinib hydrochloride at 1 × MIC, 2 × MIC, and 4 × MIC. Statistical analyses are performed using one-way analysis of variance (ANOVA) followed by Tukey’s multiple-comparisons test. Data are presented as mean ± SD from three biological replicates. ns indicates no significant difference; ** *p* < 0.01, *** *p* < 0.001.

To further evaluate the antibacterial activity of ponatinib hydrochloride against *S. aureus*, its effects on a standard susceptible strain, an MRSA strain, and a clinical multidrug-resistant *S. aureus* isolate were further investigated. As shown in Figure 2, ponatinib hydrochloride exhibited pronounced concentration-dependent antibacterial activity against *S. aureus*. In the dose–response assays, the growth of *S. aureus* ATCC 29213, MRSA ATCC 43300, and the clinical isolate 11413 was significantly inhibited as the concentration of ponatinib hydrochloride increased (Figure 2A–C). Subsequently, bacterial growth curves were analyzed to further evaluate the effects of ponatinib hydrochloride on bacterial growth kinetics. The control groups displayed a typical logarithmic growth pattern, whereas bacterial growth was markedly suppressed following ponatinib hydrochloride treatment (Figure 2D–F). At 0.5 × MIC, the bacterial growth rate was already significantly reduced; at 1 × MIC, bacterial proliferation was almost completely inhibited; and at 2 × MIC, the OD600 values of all strains remained at extremely low levels throughout the experiment, suggesting that ponatinib hydrochloride could continuously suppress bacterial growth and exhibited strong antibacterial stability (Figure 2D–F). Furthermore, time-kill curve analysis demonstrated that ponatinib hydrochloride exerted clear time- and concentration-dependent bactericidal activity. At 1 × MIC, bacterial counts remained at low levels or decreased gradually over time (Figure 2G–I). Under 2 × MIC treatment, viable bacterial counts progressively declined, whereas at 4 × MIC, the colony-forming units (CFUs) of ATCC 29213, ATCC 43300, and the clinical isolate 11413 decreased to nearly the detection limit within 24 h, indicating that ponatinib hydrochloride could rapidly and effectively eradicate bacteria (Figure 2G–I). Collectively, these results provide experimental evidences supporting its potential as a candidate agent against drug-resistant *S. aureus*.

### 2.3. Ponatinib Hydrochloride Inhibits MRSA Biofilm Formation and Disrupts Mature Biofilm Structures

**Figure 3:**
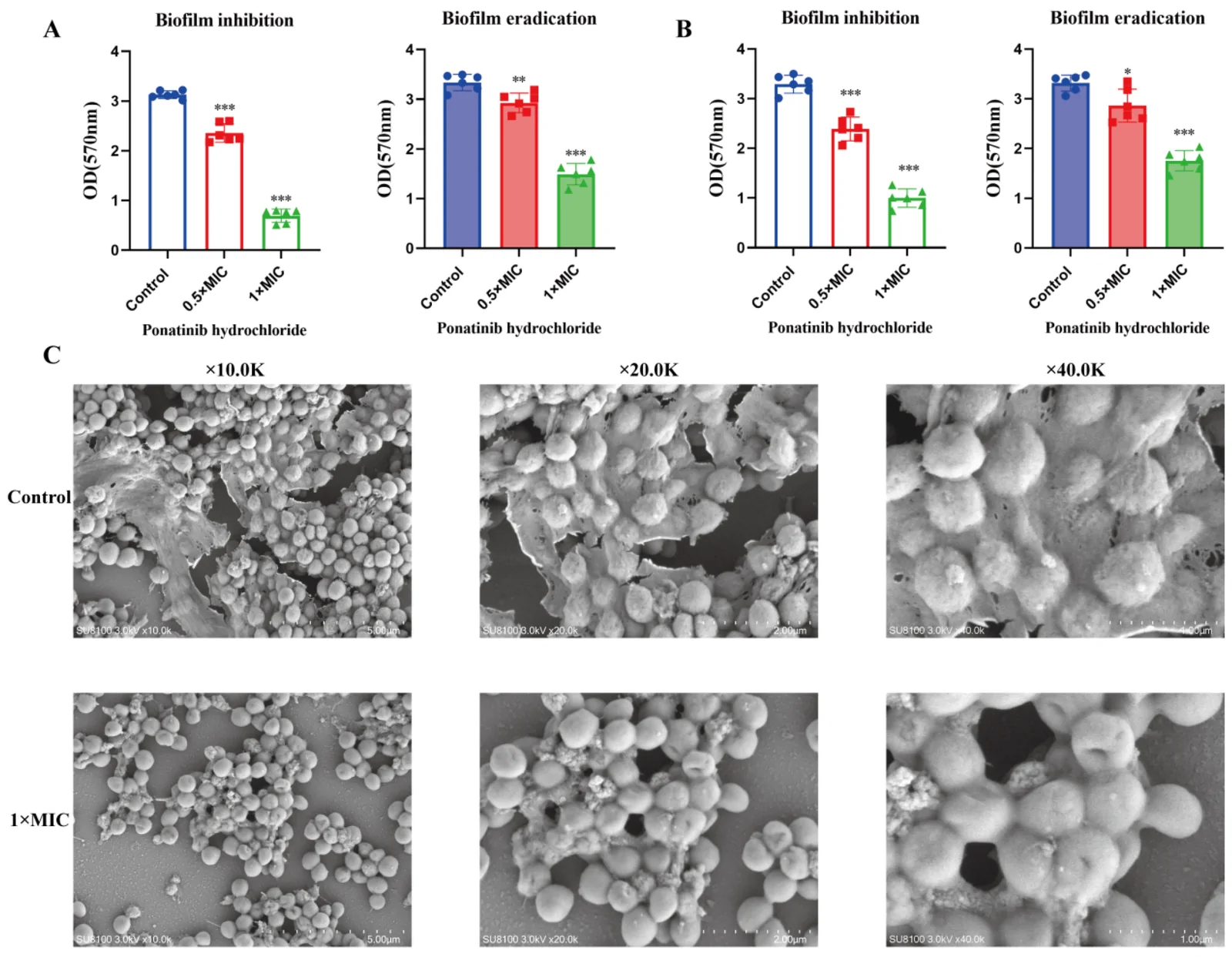
Effects of ponatinib hydrochloride on biofilm formation and mature biofilms of MSSA ATCC 29213 and MRSA ATCC 43300. (**A**,**B**) Effects of ponatinib hydrochloride at 0.5 × MIC and 1 × MIC on biofilm formation and mature biofilms of MSSA ATCC 29213 and MRSA ATCC 43300, with biofilm biomass quantified by OD570 values. (**C**) Morphological changes in MRSA ATCC 43300 biofilms following ponatinib hydrochloride treatment observed by scanning electron microscopy (SEM). The control group was untreated, whereas the experimental group was treated with 1 × MIC ponatinib hydrochloride. Biofilm structures are shown at magnifications of 10.0 K, 20.0 K, and 40.0 K. Statistical analyses are performed using one-way analysis of variance (ANOVA) followed by Tukey’s multiple-comparisons test. Data are presented as mean ± SD. * *p* < 0.05, ** *p* < 0.01, *** *p* < 0.001.

To further evaluate the effects of ponatinib hydrochloride on *S. aureus* biofilms, the MSSA reference strain ATCC 29213 and MRSA reference strain ATCC 43300 were used to investigate its influence on biofilm formation and mature biofilm eradication. The results demonstrated that ponatinib hydrochloride treatment markedly reduced the biofilm biomass of *S. aureus* in a concentration-dependent manner during the biofilm formation assay (Figure 3A,B). When the drug concentration increased from 0.5 × MIC to 1 × MIC, the OD570 values decreased further, indicating that ponatinib hydrochloride effectively inhibited MRSA biofilm formation (Figure 3A,B). Similarly, in the mature biofilm eradication assay, ponatinib hydrochloride also reduced the biomass of preformed mature biofilms, with a more pronounced reduction observed in the 1 × MIC treatment group (Figure 3A,B). These findings suggest that ponatinib hydrochloride not only interferes with biofilm formation but also exerts disruptive effects on established mature biofilms. Further morphological changes in MRSA 43300 biofilms were examined by scanning electron microscopy (SEM). In the control group, bacteria were densely aggregated and formed compact and intact biofilm structures with abundant clustered architectures (Figure 3C). In contrast, following treatment with 1 × MIC ponatinib hydrochloride, bacterial aggregation was markedly reduced, the biofilm structure became loose and disorganized, and the connections between bacterial cells were decreased, with obvious gaps observed in some regions (Figure 3C). These observations indicate that ponatinib hydrochloride can significantly disrupt the structural integrity of *S. aureus* biofilms.

### 2.4. Drug Resistance Induction and Preliminary Safety Evaluation of Ponatinib Hydrochloride

**Figure 4:**
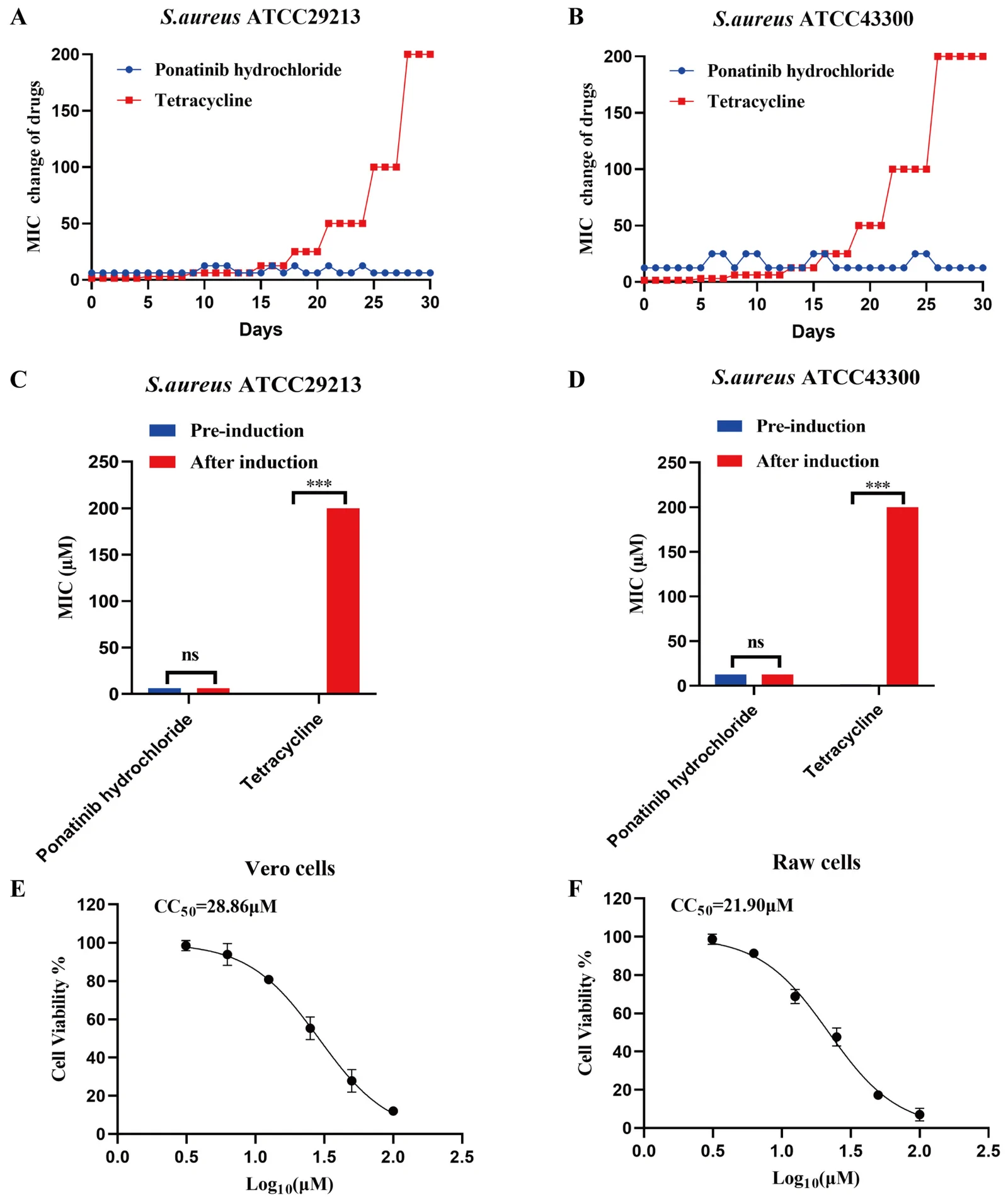
Effects of ponatinib hydrochloride on MIC changes in *S. aureus* during serial passaging. (**A**) Changes in MIC values of *S. aureus* ATCC 29213 during serial passaging with ponatinib hydrochloride and tetracycline treatment. (**B**) Changes in MIC values of MRSA ATCC 43300 during serial passaging with ponatinib hydrochloride and tetracycline treatment. The *x*-axis represents the number of serial passaging days, and the *y*-axis represents the fold change in MIC values (MIC change of drugs). Changes in the MICs of ponatinib hydrochloride and tetracycline for *S. aureus* ATCC 29213 (**C**) and MRSA ATCC 43300 (**D**) before and after drug induction. Effect of ponatinib hydrochloride on the viability of Vero (**E**) and RAW 264.7 (**F**) cells. Statistical analysis was performed using an unpaired two-tailed Student’s *t*-test. Data are presented as mean ± SD. ns indicates no significant difference; *** *p* < 0.001.

To further evaluate the risk of resistance development induced by ponatinib hydrochloride, serial passaging experiments were performed to monitor changes in the MICs of MSSA 29213 and MRSA ATCC 43300 under prolonged drug pressure, with tetracycline used as the control antibiotic. As shown in Figure 3, during the 30-day serial passaging period, the MICs of the ponatinib hydrochloride-treated groups remained relatively stable, exhibiting only slight fluctuations without any obvious persistent upward trend. In contrast, the tetracycline-treated groups exhibited a continuous increase in MIC values during serial passaging. As the passaging period progressed, the MICs of tetracycline against both strains increased markedly and reached substantially elevated levels at later stages, indicating that bacteria were more prone to developing resistance under tetracycline pressure (Figure 4A,B). To further assess whether the sensitivity changes observed during continuous passage can be stably maintained after the removal of drug pressure, the strain was further passaged for 5 consecutive generations in a drug-free medium. The results showed that ponatinib hydrochloride did not exhibit significant fluctuations in MIC for *S. aureus* ATCC29213 and ATCC43300 (Table 2). Meanwhile, after the removal of drug pressure, the sensitivity of the strain to tetracycline did not show a stable change, indicating that the MIC fluctuations generated during continuous passage formed a stable resistance phenotype (Table 2, Figure 4C,D). Furthermore, the evaluation of the cytotoxicity of ponatinib hydrochloride against Vero and RAW cells revealed that the survival rates of both cell types gradually decreased as the drug concentration increased, demonstrating a clear concentration-dependent effect. The CC_50_ values for Vero and RAW cells were 28.86 μM and 21.90 μM (Figure 4E,F), respectively, indicating that ponatinib hydrochloride exhibited relatively low cytotoxicity at concentrations below the CC_50_.

### 2.5. Ponatinib Hydrochloride Affects the Survival of S. aureus by Disrupting Membrane Integrity and Inducing Oxidative Stress

**Figure 5:**
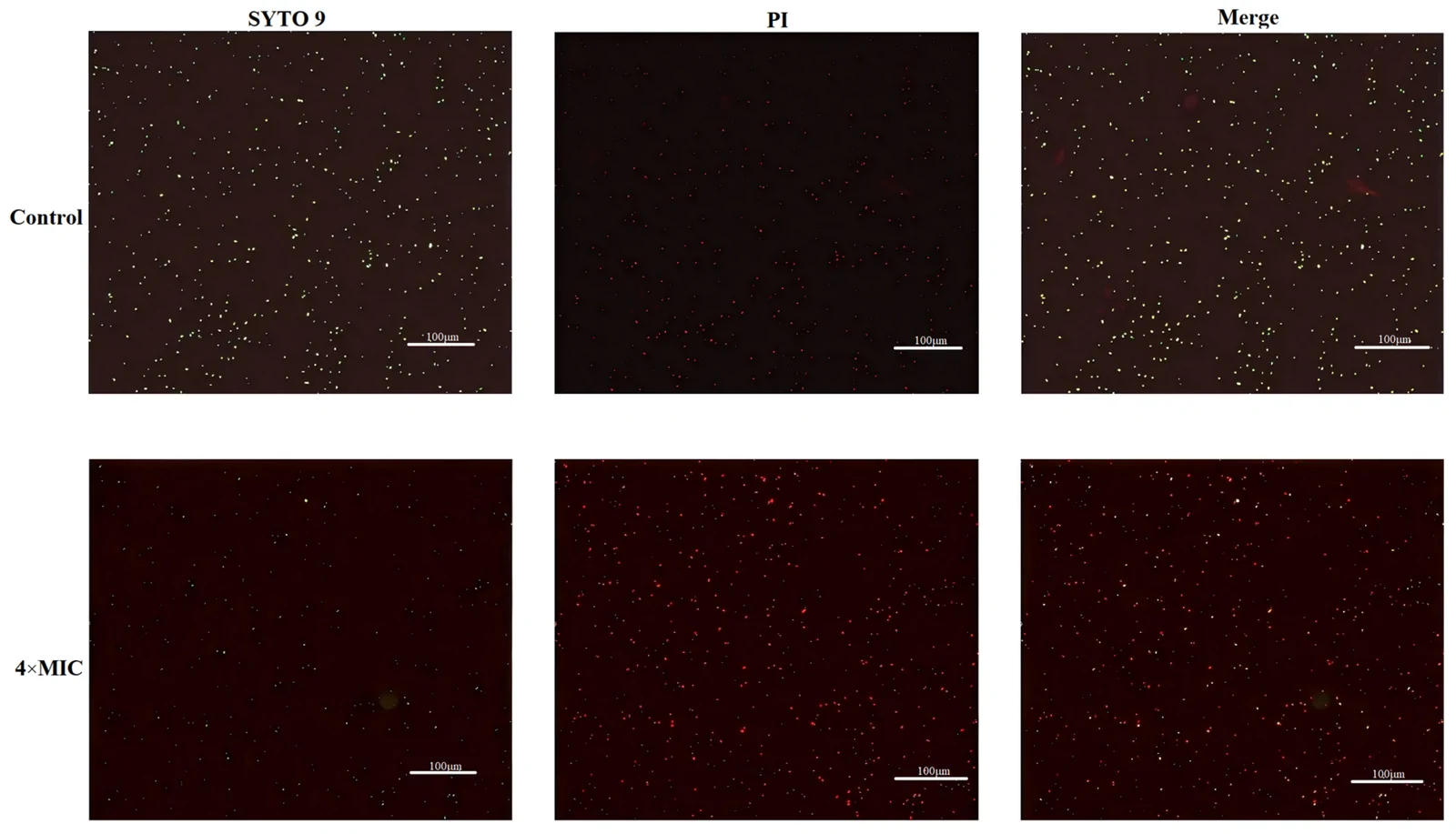
Changes in membrane integrity of *S. aureus* ATCC 29213 following ponatinib hydrochloride treatment observed by SYTO 9/PI staining. The control group was untreated, whereas the experimental group was treated with 4 × MIC ponatinib hydrochloride. Scale bar = 100 μm.

**Figure 6:**
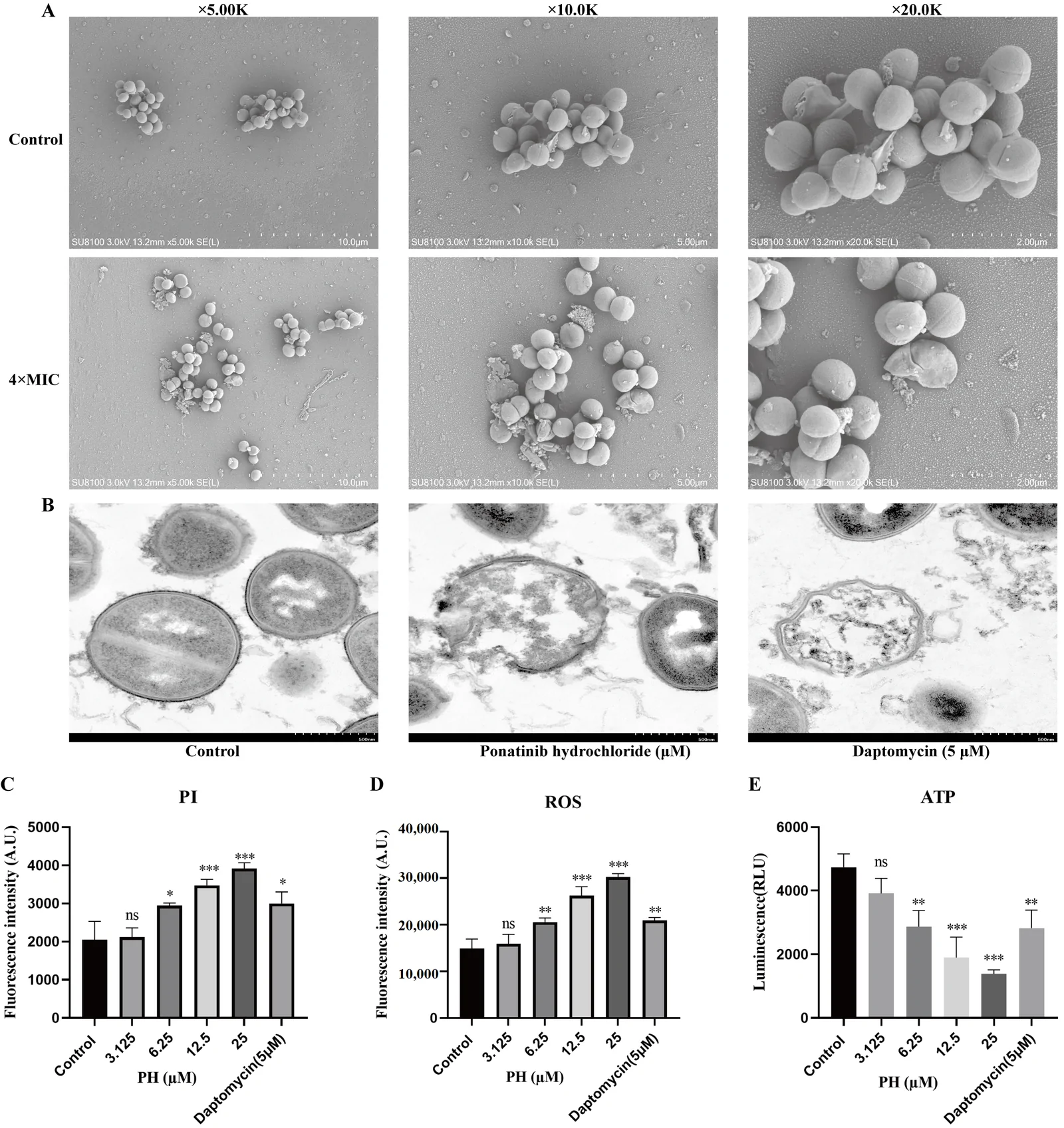
Investigation of the antibacterial mechanism of ponatinib hydrochloride. (**A**) Morphological changes in *S. aureus* ATCC 29213 following ponatinib hydrochloride treatment observed by scanning electron microscopy (SEM). The control group was untreated, whereas the experimental group was treated with 4 × MIC ponatinib hydrochloride. Bacterial morphology is shown at magnifications of 5.00 K, 10.0 K, and 20.0 K. (**B**) Transmission electron microscopy (TEM) analysis of bacterial ultrastructural changes following treatment with ponatinib hydrochloride. (**C**) Changes in PI fluorescence intensity of *S. aureus* ATCC 29213 following ponatinib hydrochloride treatment. (**D**) Changes in intracellular ROS levels of *S. aureus* ATCC 29213 following ponatinib hydrochloride treatment. (**E**) Changes in intracellular ATP levels of *S. aureus* ATCC 29213 following ponatinib hydrochloride treatment. Daptomycin (5 μM) was used as the positive control. Data are presented as mean ± SD. ns indicates no significant difference; * *p* < 0.05, ** *p* < 0.01, *** *p* < 0.001.

To further elucidate the potential antibacterial mechanism of ponatinib hydrochloride, this study further investigated its mode of action. Bacterial membrane integrity was first evaluated using SYTO 9/PI dual staining. In the control group, bacteria mainly exhibited green fluorescence, whereas treatment with 4 × MIC ponatinib hydrochloride markedly increased red fluorescence signals and significantly reduced green fluorescence, indicating substantial alterations in bacterial membrane permeability (Figure 5). Merged images further showed a pronounced increase in the proportion of red fluorescence in the treated group, suggesting that ponatinib hydrochloride significantly disrupted bacterial membrane integrity (Figure 5). Subsequently, scanning electron microscopy (SEM) was employed to further observe changes in bacterial surface morphology. The control bacteria displayed intact morphology with smooth cell surfaces and compact cellular arrangements (Figure 6A). In contrast, treatment with 4 × MIC ponatinib hydrochloride induced obvious morphological abnormalities, including cell shrinkage, collapse, and structural damage in bacterial cells, while bacterial aggregation was also disrupted, indicating that ponatinib hydrochloride caused substantial damage to bacterial cellular structures (Figure 6A). Furthermore, transmission electron microscopy revealed that the bacterial cell membranes and cytoplasmic structures in the control group remained intact; following treatment with ponatinib hydrochloride, the bacteria exhibited distinct ultrastructural alterations, including damage to the cell membrane structure, loosening of the cytoplasm, and an increase in electron-lucent regions; the daptomycin group likewise displayed significant disruption of the membrane structure and cytoplasmic disarray (Figure 6B). Further analysis of membrane damage-related fluorescence revealed that PI fluorescence intensity gradually increased with increasing concentrations of ponatinib hydrochloride, with more pronounced elevation observed at concentrations of 6.25–25 μM, indicating enhanced membrane permeability (Figure 6C). High-PI fluorescence signals were similarly observed in the daptomycin-treated positive control group (Figure 6C). In addition, ROS assays demonstrated that intracellular ROS levels significantly increased following ponatinib hydrochloride treatment in a concentration-dependent manner, with the most prominent ROS accumulation detected in the 12.5 μM and 25 μM treatment groups (Figure 6D). Meanwhile, ATP assays showed that intracellular ATP levels markedly decreased after ponatinib hydrochloride treatment and exhibited a concentration-dependent downward trend, with the most significant reduction observed in the 25 μM treatment group (Figure 6E). These findings indicate that ponatinib hydrochloride interferes with bacterial energy metabolism. In short, these results demonstrate that ponatinib hydrochloride exerts antibacterial activity through multiple methods.

### 2.6. Ponatinib Hydrochloride Exhibits Protective Effects Against Clinical Multidrug-Resistant S. aureus in Mouse Infection Models

**Figure 7:**
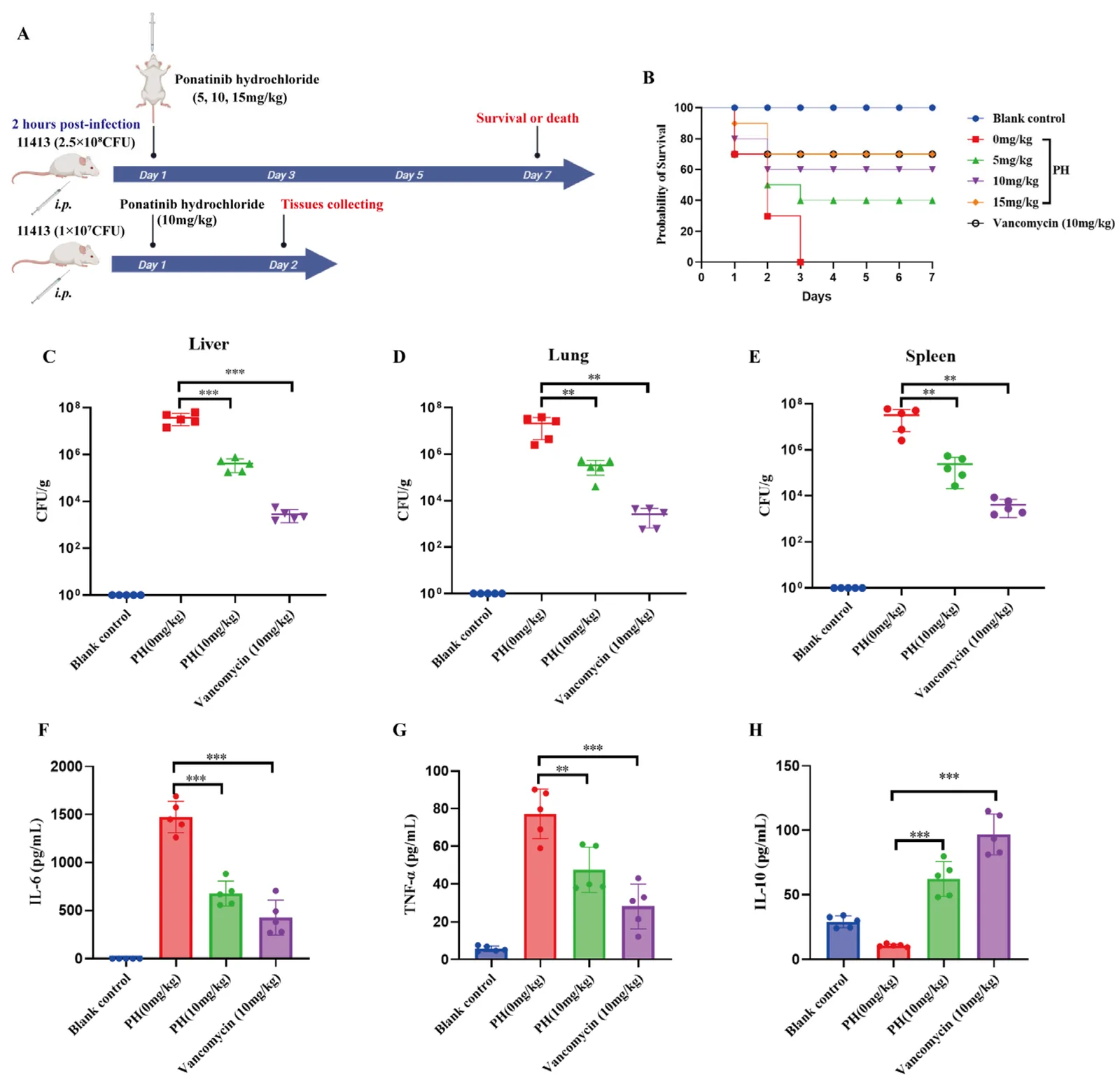
Protective effects of ponatinib hydrochloride against clinical multidrug-resistant *S. aureus* 11413 infection in mice. (**A**) Schematic illustration of the lethal and non-lethal mouse intraperitoneal infection models. The lethal infection model was used to evaluate mouse survival, whereas the non-lethal infection model was used to assess tissue bacterial burdens and inflammatory cytokine levels. (**B**) Seven-day survival curves of infected mice following ponatinib hydrochloride treatment. (**C**–**E**) Changes in bacterial burdens in the liver, lungs, and spleen of mice following ponatinib hydrochloride treatment, expressed as CFU/g. (**F**–**H**) Changes in serum IL-6, TNF-α, and IL-10 levels in mice following ponatinib hydrochloride treatment. Data are presented as mean ± SD. ** *p* < 0.01, *** *p* < 0.001.

To further evaluate the in vivo antibacterial activity of ponatinib hydrochloride against clinical multidrug-resistant *S. aureus*, lethal and non-lethal mouse intraperitoneal infection models were established to assess its effects on infection host. As shown in Figure 6A, in the lethal infection model, mice were intraperitoneally challenged with the clinical multidrug-resistant *S. aureus* isolate 11413 (2.5 × 10^8^ CFU) and treated with ponatinib hydrochloride at doses of 5, 10, or 15 mg/kg at 2 h post-infection, followed by survival monitoring for 7 days. In the non-lethal infection model, mice were intraperitoneally infected with 1 × 10^7^ CFU bacteria and treated with ponatinib hydrochloride (10 mg/kg), and tissues were collected on day 2 post-infection for bacterial burden and inflammatory cytokine analyses. Survival curve analysis demonstrated that all mice in the infected group died within 3 days post-infection, resulting in a final survival rate of 0% (Figure 7B). In contrast, ponatinib hydrochloride treatment significantly improved mouse survival in a dose-dependent manner. The final survival rates were 40%, 60%, and 70% in the 5, 10, and 15 mg/kg treatment groups, respectively, with the protective efficacy of the 15 mg/kg group comparable to that of the vancomycin-treated group (10 mg/kg, 70%) (Figure 7B). Further analysis of bacterial burdens in the liver, lungs, and spleen revealed that ponatinib hydrochloride treatment markedly reduced bacterial loads compared with the infected group (Figure 7C–E). In particular, the 10 mg/kg treatment group exhibited significantly lower CFU counts in the liver, lungs, and spleen, indicating that ponatinib hydrochloride effectively reduced bacterial colonization and dissemination in vivo (Figure 7C–E). The vancomycin-treated group showed even lower bacterial burden levels. In addition, inflammatory cytokine analysis showed that infection markedly increased the levels of IL-6 and TNF-α in mice, whereas ponatinib hydrochloride treatment significantly reduced the levels of these pro-inflammatory cytokines (Figure 7F,G). Meanwhile, the level of the anti-inflammatory cytokine IL-10 was significantly elevated following treatment, suggesting that ponatinib hydrochloride could modulate infection-associated inflammatory responses while reducing bacterial burdens (Figure 7H). These results demonstrate that ponatinib hydrochloride exhibits in vivo antibacterial activity and host-protective effects in mouse models of clinical multidrug-resistant *S. aureus* infection.

## 3. Discussion

With the continuous prevalence of methicillin-resistant *S. aureus* (MRSA) and multidrug-resistant *S. aureus*, the declining efficacy of conventional antibiotics has become a major challenge in global public health [3]. Meanwhile, the development of conventional antibiotics has markedly lagged behind the rapid evolution of bacterial resistance, further highlighting the value of drug repurposing strategies in anti-infective research [15]. Ponatinib hydrochloride, an FDA-approved small-molecule tyrosine kinase inhibitor, was originally developed for the treatment of drug-resistant leukemia, and its pharmacokinetics and safety profiles have been extensively investigated in the field of oncology [19]. However, its antibacterial activity has not yet been systematically evaluated. In the present study, we demonstrated that ponatinib hydrochloride possessed antibacterial activity against multidrug-resistant *S. aureus*, thereby expanding the potential application value of this class of targeted agents in the field of anti-infective therapy.

In this study, ponatinib hydrochloride exhibited antibacterial activity against Gram-positive bacteria, whereas its activity against Gram-negative bacteria such as *E. coli* and *P. aeruginosa* was relatively weak. This difference may be associated with the outer membrane barrier of Gram-negative bacteria, which restricts drug penetration [21]. Previous studies have shown that many hydrophobic small-molecule compounds preferentially act against Gram-positive bacteria but are less capable of penetrating the lipopolysaccharide outer membrane of Gram-negative bacteria [22,23]. Notably, ponatinib hydrochloride not only displayed stable activity against standard MRSA strains but also maintained low MIC values against multiple clinical multidrug-resistant isolates. Compared with previously reported drug repurposing studies, such as those involving doxorubicin [24,25] or certain nonsteroidal anti-inflammatory drugs [26], which generally require relatively high concentrations to achieve effective anti-MRSA activity and often exhibit unstable efficacy against clinical resistant isolates. Ponatinib hydrochloride inhibited MRSA growth at low micromolar concentrations, suggesting better antibacterial potency. Further time-kill analyses demonstrated that ponatinib hydrochloride exerted concentration-dependent and time-dependent bactericidal activity, which provided an experimental basis for further research into its potential as an antibacterial drug candidate.

MRSA biofilm formation is one of the major causes of chronic infections, implant-associated infections, and antibiotic treatment failure [27]. In the present study, ponatinib hydrochloride not only inhibited MRSA biofilm formation but also exhibited pronounced disruptive effects on mature biofilms. SEM observations further confirmed that bacterial aggregation structures were markedly reduced after treatment, and the biofilm architecture became loose and disorganized. Previous studies have demonstrated that many conventional antibiotics possess limited ability to eradicate mature biofilms, and even first-line agents such as vancomycin often fail to completely penetrate established biofilm structures [28]. Therefore, the dual activity of ponatinib hydrochloride against both planktonic bacteria and biofilms is of particular interest. In recent years, several natural products and membrane-active compounds, including curcumin [29], thymol [30], and daptomycin [31], have also been reported to affect MRSA biofilm stability. The findings of this study suggest that ponatinib hydrochloride similarly disrupts biofilm structural integrity. Notably, 30-day serial passaging experiments demonstrated that ponatinib hydrochloride did not induce a sustained increase in MIC values under drug pressure, whereas tetracycline rapidly induced resistance development. However, current results indicate only that no obvious phenotypic resistance to ponatinib hydrochloride was observed under continuous drug pressure; they are insufficient to confirm the occurrence of resistance or its underlying mechanisms at the molecular level. Further experimental verification is still needed in the future. Moreover, while the tendency of ponatinib hydrochloride to induce drug resistance offers value for further development, the proximity of its antimicrobial concentrations to cytotoxic levels suggests that safety remains a critical factor limiting clinical translation. Therefore, future research should aim to further reduce host cytotoxicity—through structural modification or combination therapy strategies—while maintaining antimicrobial activity, thereby broadening its potential therapeutic window.

Mechanistic investigations further demonstrated that ponatinib hydrochloride markedly disrupted bacterial membrane integrity. As the bacterial membrane is essential for maintaining osmotic balance, energy metabolism, and material transport, membrane damage can rapidly result in the disruption of cellular homeostasis [32]. Increasing evidence suggests that, compared with agents targeting a single intracellular pathway, membrane-disruptive mechanisms are less susceptible to resistance development through single-point mutations [33,34]. In addition, this study demonstrated that ponatinib hydrochloride induced substantial intracellular ROS accumulation accompanied by a marked reduction in ATP levels. Previous studies have shown that excessive ROS accumulation can further trigger lipid peroxidation, protein damage, and DNA damage, thereby accelerating bacterial cell death [35], whereas ATP depletion indicates severe disruption of bacterial energy metabolism [36]. Notably, previous studies have proposed that Ponatinib may possess the potential to target the bacterial cell division protein FtsZ [20]. As a key protein responsible for Z-ring formation during bacterial cytokinesis, FtsZ is considered an essential target for bacterial cell division [37]. Although the present study did not directly verify the effect of ponatinib hydrochloride on *S. aureus* FtsZ, its low tendency to induce resistance together with the multiple cellular damage phenotypes observed suggest that its antibacterial mechanism is unlikely to rely on a single mode of action.

In addition to its in vitro antibacterial activity, the present study further demonstrated that ponatinib hydrochloride exerted significant host-protective effects in murine infection models. Previous studies have shown that host mortality during severe *S. aureus* infections, particularly sepsis, is associated not only with bacterial burden but also with cytokine storms and immune dysregulation [38]. Therefore, small-molecule agents possessing both antibacterial and immunomodulatory activities may provide additional advantages in infection therapy. Considering that Ponatinib, as a tyrosine kinase inhibitor, is known to possess immunomodulatory properties, the anti-inflammatory effects observed in the infection models may not solely result from bacterial clearance but may also involve the regulation of host signaling pathways [39].

### Limitations

Although this study evaluated the antibacterial activity and mechanisms of ponatinib hydrochloride against multidrug-resistant *S. aureus*, several limitations remain. First, although its antibacterial activity appears to involve membrane damage, ROS accumulation, and disruption of energy metabolism, the direct molecular targets (FtsZ) and underlying mechanisms remain to be further clarified. Second, as an approved tyrosine kinase inhibitor, Ponatinib has been reported to carry certain cardiovascular toxicity risks in anticancer applications [40]; therefore, its systemic safety, tissue distribution, and pharmacokinetic properties under anti-infective dosing conditions require further evaluation. Finally, this study did not evaluate the combination of ponatinib hydrochloride with conventional antibiotics, which may further reduce resistance development and enhance therapeutic efficacy.

## 4. Materials and Methods

### 4.1. Bacterial Strains, Culture Conditions, and Drug Preparation

The standard strains used in this study included *S. aureus* ATCC 29213, methicillin-resistant *S. aureus* (MRSA) ATCC 43300, *Streptococcus pneumoniae* ATCC 49619, *Escherichia coli* ATCC 25922, and *Pseudomonas aeruginosa* ATCC 9027. In addition, 15 clinical multidrug-resistant *S. aureus* isolates preserved in our laboratory were collected for antimicrobial susceptibility testing. The clinical isolates were derived from tissue samples associated with clinical infections in animals at the Ruoxi Specialized Breeding Cooperative in Yuncheng County, Heze, Shandong Province, China; these samples included infected organ tissues, abscesses, and wounds. All strains were stored in bacterial preservation solution containing 30% glycerol at −80 °C. Prior to experiments, bacterial strains were streaked onto Mueller–Hinton agar (MHA) plates and cultured at 37 °C for 18–24 h. Single colonies were then inoculated into Mueller–Hinton broth (MHB) and cultured at 37 °C with shaking at 200 rpm until reaching the logarithmic growth phase (OD600 = 0.6–0.8). For *S. pneumoniae*, the MHB culture medium was supplemented with 5% fetal bovine serum. Ponatinib hydrochloride was purchased from MedChemExpress (MCE, Monmouth Junction, NJ, USA), and dissolved in sterile dimethyl sulfoxide (DMSO) to prepare a 20 mM stock solution, which was stored at −20 °C protected from light. Prior to use, the stock solution was serially diluted with MHB, and the final DMSO concentration in all experimental systems was maintained below 1%. Tetracycline, daptomycin, and vancomycin were purchased from Shanghai Aladdin Biochemical Technology, China. The mouse macrophage cell line RAW264.7 and African green monkey kidney epithelial cell line Vero cells were cultured in Dulbecco’s Modified Eagle Medium (DMEM; Gibco, Thermo Fisher Scientific, Waltham, MA, USA) supplemented with 10% fetal bovine serum (FBS; Gibco, Thermo Fisher Scientific, Waltham, MA, USA) and 1% penicillin–streptomycin at 37 °C in a humidified incubator containing 5% CO_2_.

### 4.2. Determination of Minimum Inhibitory Concentration (MIC)

The minimum inhibitory concentrations (MICs) of ponatinib hydrochloride against different bacterial strains were determined using the broth microdilution method in cation-adjusted Mueller–Hinton broth (CAMHB) (Ca^2+^, 20 mg/L; Mg^2+^, 10 mg/L) according to the guidelines of the Clinical and Laboratory Standards Institute (CLSI) [41]. Bacteria in the logarithmic growth phase were collected by centrifugation (5000× *g*, 10 min), washed twice with sterile PBS, and adjusted to a 0.5 McFarland standard (approximately 1 × 10^8^ CFU/mL), followed by further dilution to approximately 1 × 10^6^ CFU/mL. In sterile 96-well plates, 100 μL of bacterial suspension and 100 μL of ponatinib hydrochloride working solution at different concentrations were added to each well, resulting in final drug concentrations ranging from 0.78 to 100 μM. The final concentration of the bacterial suspension was 5 × 10^5^ CFU/mL. Tetracycline was used as the positive control for the antibiotic. Medium blank controls, bacterial growth controls, and DMSO solvent controls (0.5%) were included in parallel. After incubation at 37 °C for 18 h under static conditions, bacterial growth was evaluated by visual observation. The MIC was defined as the lowest drug concentration at which no visible bacterial growth was observed. The experiments were performed with three independent replicates.

### 4.3. Dose–Response Assay

Bacteria in the logarithmic growth phase were diluted with MHB to approximately 1 × 10^6^ CFU/mL and added to 96-well plates with a final volume of 200 μL per well. Ponatinib hydrochloride was then added at different concentrations ranging from 0.78 to 100 μM. The plates were incubated at 37 °C for 18 h, after which OD600 values were measured using a multifunctional microplate reader, and dose–response curves were generated [42].

### 4.4. Bacterial Growth Curve Assay

Bacteria in the logarithmic growth phase were diluted to approximately 1 × 10^6^ CFU/mL and cultured in MHB containing 0.5 × MIC, 1 × MIC, or 2 × MIC ponatinib hydrochloride. The cultures were incubated at 37 °C with shaking at 200 rpm, and OD600 values were measured every 2 h using a multifunctional microplate reader for a total duration of 24 h. Bacterial growth curves were generated based on the changes in OD600 values [43].

### 4.5. Time-Kill Curve Assay

*S. aureus* ATCC 29213, MRSA ATCC 43300, and the clinical isolate 11413 were diluted to approximately 1 × 10^6^ CFU/mL and incubated with ponatinib hydrochloride at concentrations of 1 × MIC, 2 × MIC, and 4 × MIC. At 0, 3, 6, 9, 12, and 24 h, 100 μL bacterial suspensions were collected and subjected to 10-fold serial dilutions with sterile PBS. Subsequently, 100 μL of each diluted suspension was spread onto MHA plates and incubated at 37 °C for 18 h. Colony-forming units (CFUs) were then counted and expressed as CFU/mL [44]. At each time point, bacterial suspensions underwent serial gradient dilution and were plated for culture; colony-forming units (CFUs) were counted after incubation, and bacterial survival was expressed as CFU/mL. The limit of detection (LOD) for bacterial enumeration under the experimental conditions was determined to be 1 CFU/mL. Samples with no detectable colonies were assigned a value of 1 CFU/mL for subsequent statistical analysis.

### 4.6. Biofilm Formation Inhibition Assay

The effects of ponatinib hydrochloride on MRSA biofilm formation were evaluated using a crystal violet staining assay [45]. MSSA ATCC 29213 and MRSA ATCC 43300 were cultured in TSB medium to the logarithmic growth phase and diluted to approximately 1 × 10^6^ CFU/mL using TSB supplemented with 1% glucose. Subsequently, 100 μL of bacterial suspension and 100 μL of ponatinib hydrochloride working solution at different concentrations were added to each well of a 96-well plate. For MSSA ATCC 29213, the final drug concentrations were 3.125µM and 6.25µM, respectively. For MRSA ATCC 43300, the final drug concentrations were 6.25µM and 12.5µM, respectively. After static incubation at 37 °C for 24 h, the culture medium was carefully removed, and the wells were gently washed three times with sterile PBS to eliminate planktonic bacteria. Biofilms were fixed with 200 μL methanol per well for 15 min and then stained with 200 μL of 0.1% crystal violet for 15 min. After washing with PBS to remove unbound dye, the plates were air-dried at room temperature. Finally, 200 μL of 95% ethanol was added to dissolve the bound crystal violet, and the absorbance was measured at 570 nm (OD570).

### 4.7. Mature Biofilm Eradication Assay

Based on the procedures used in the biofilm formation inhibition assay, MRSA suspensions were statically incubated in 96-well plates at 37 °C for 24 h to allow for the formation of mature biofilms. After removal of the culture medium and washing three times with PBS, fresh medium containing different concentrations of ponatinib hydrochloride was added, followed by an additional 24 h treatment. Similarly, the final concentrations of ponatinib hydrochloride were 0.5 × MIC and 1 × MIC, respectively. The residual mature biofilm biomass was then quantified using the crystal violet staining method described above.

### 4.8. Scanning Electron Microscopy (SEM) and Transmission Electron Microscopy (TEM) Observations

Based on the biofilm culture procedure, sterile circular coverslips were placed into 24-well plates, followed by the addition of 1 mL bacterial suspension and the corresponding concentrations of ponatinib hydrochloride to each well. After incubation at 37 °C for 24 h, the culture medium was discarded, and the samples were gently washed three times with PBS. The samples were fixed with 2.5% glutaraldehyde at 4 °C overnight and then sequentially dehydrated using graded ethanol solutions of 30%, 50%, 70%, 80%, 90%, and 100% for 10 min each. Subsequently, the samples were replaced with tert-butanol and freeze-dried. After drying, the samples were sputter-coated with gold and finally observed using scanning electron microscopy to evaluate changes in bacterial and biofilm morphology.

The processing methods of TEM for the bacterial samples were identical to those used for scanning electron microscopy. Bacterial pellets were collected via centrifugation (with a pellet volume at least the size of a mung bean); the culture medium was discarded, and an electron microscopy fixative was added. The samples were resuspended and fixed at 4 °C for 2–4 h, then stored and transported in the dark. After centrifugation, the samples were rinsed three times (3 min each) with 0.1 M phosphate buffer (PB, pH 7.4) and subsequently pre-embedded in pre-warmed, dissolved 1% agarose. The samples were fixed in 1% osmium tetroxide (prepared in 0.1 M PB, pH 7.4) at room temperature in the dark for 2 h, followed by three rinses in PB (15 min each). Dehydration was performed using a graded ethanol series (30%, 50%, 70%, 80%, 95%, and 100%) for 20 min per step, followed by two changes of 100% acetone (15 min each). Infiltration was carried out sequentially using mixtures of acetone and 812 embedding resin at ratios of 1:1 and 1:2 at 37 °C (2–4 h and overnight, respectively), followed by infiltration with pure 812 resin at 37 °C for 5–8 h. The samples were then placed in embedding molds and polymerized at 37 °C overnight, followed by final polymerization at 60 °C for 48 h. Ultrathin sections (60–80 nm) were prepared from the resin blocks using an ultramicrotome and collected on 150-mesh copper grids coated with Formvar film. The sections were stained with a saturated alcoholic solution of 2% uranyl acetate for 8 min in the dark, rinsed three times each with 70% ethanol and ultrapure water, stained with 2.6% lead citrate solution for 8 min (protected from CO_2_), rinsed three times with ultrapure water, and dried at room temperature overnight. Finally, the samples were examined and imaged using a transmission electron microscope (HT7800, Hitachi, Tokyo, Japan) for analysis.

### 4.9. Serial Passaging Resistance Induction Assay

*S. aureus* ATCC 29213 and MRSA ATCC 43300 were inoculated into MHB containing 0.5 × MIC ponatinib hydrochloride and incubated at 37 °C for 18 h. The cultures were then transferred at a 1:100 ratio into fresh drug-containing medium for continued incubation, and MIC values were re-determined daily. Bacterial strains were continuously cultured in medium containing 0.5× MIC of ponatinib hydrochloride. The drug concentration in the culture medium was adjusted based on the latest MIC determination values. This procedure was continuously performed for 30 days. Tetracycline was used as the positive control drug. Changes in MIC values were recorded based on the MIC measured each day [46]. To further evaluate bacterial stability following drug withdrawal, *S. aureus* strains subjected to serial passage were transferred to drug-free medium and cultured for an additional five generations; the MICs of ponatinib hydrochloride and tetracycline were determined for each generation.

### 4.10. Cell Counting Kit-8 (CCK-8) Assay

The CCK-8 assay was used to evaluate the effect of ponatinib hydrochloride on the viability of Vero and RAW 264.7 cells. Cells were cultured to the logarithmic growth phase, trypsinized to prepare a single-cell suspension, and seeded into 96-well plates at an appropriate density (100 μL per well). Experimental groups included a blank control (DMEM control), a cell control (untreated cell group), and groups treated with various concentrations of ponatinib hydrochloride. After the cells adhered, ponatinib hydrochloride was added at different concentrations, and the cells were cultured for an additional 24 h. Following treatment, 10 μL of CCK-8 reagent was added to each well, and the plates were incubated at 37 °C with 5% CO_2_ for 1–2 h. Absorbance at 450 nm (OD_450_) was then measured using a microplate reader. Using the untreated cell group as the control, relative cell viability was calculated using the following formula: Cell viability (%) = (OD_treated − OD_blank)/(OD_control − OD_blank) × 100%. At least three replicate wells were set up for each group, and the experiment was independently repeated three times. Cytotoxicity was evaluated based on changes in cell viability following treatment with various concentrations of ponatinib hydrochloride, and a concentration–effect curve was fitted using non-linear regression to calculate the 50% cytotoxic concentration (CC_50_).

### 4.11. SYTO 9/PI Dual-Staining Assay

Bacterial membrane integrity was evaluated using the LIVE/DEAD BacLight bacterial viability kit [47]. Based on the time-kill assay results, 4 MIC was selected for the SYTO 9/PI assay because it produced bactericidal effect within 2 h, allowing for rapid assessment of changes in bacteria. Bacteria in the logarithmic growth phase were adjusted to an OD600 of approximately 0.5 and incubated with 4 × MIC ponatinib hydrochloride for 2 h. The bacterial cells were then collected by centrifugation and washed twice with PBS. Subsequently, SYTO 9 and PI mixed staining solution was added, followed by incubation at room temperature in the dark for 15 min. Finally, 10 μL of bacterial suspension was placed onto glass slides, and the changes in green and red fluorescence were observed using a fluorescence microscope.

### 4.12. PI Uptake Assay

The PI uptake assay was performed based on previously reported methods with minor modifications [48]. Bacteria in the logarithmic growth phase were adjusted to an OD600 of approximately 0.5 and incubated with different concentrations of ponatinib hydrochloride for 2 h. Subsequently, PI working solution was added to a final concentration of 10 μg/mL, followed by incubation in the dark for an additional 15 min. Fluorescence intensity was measured using a multifunctional microplate reader with excitation and emission wavelengths set at 535 nm and 617 nm, respectively. Daptomycin was used as the positive control. Untreated bacteria served as the negative control, while sterile buffer plus PI was used as the blank.

### 4.13. Intracellular ROS Detection Assay

Intracellular ROS levels were determined using the DCFH-DA fluorescent probe [47]. After treatment with different concentrations of ponatinib hydrochloride, bacteria were incubated with DCFH-DA at a final concentration of 10 μM at 37 °C in the dark for 30 min. The cells were then washed three times with PBS, and fluorescence intensity was measured using a multifunctional microplate reader with excitation and emission wavelengths of 488 nm and 525 nm, respectively. Daptomycin was used as the positive control. Untreated bacteria served as the negative control. Three independent biological replicates were performed for each experimental group (*n* = 3).

### 4.14. Intracellular ATP Measurement Assay

Intracellular ATP levels were measured using an ATP bioluminescence assay kit [49]. Bacteria in the logarithmic growth phase were adjusted to an OD600 of approximately 0.5 and incubated with different concentrations of ponatinib hydrochloride for 2 h. Bacterial suspensions were centrifuged at 12,000× *g* for 10 min at 4 °C to collect the bacterial pellets, and fully lysed using ATP lysis buffer. Subsequently, ATP detection reagent was added, and luminescence intensity was measured using a multifunctional microplate reader.

### 4.15. Mouse Intraperitoneal Infection Model

The animal procedures used in this study were performed based on previously published methods with minor modifications as appropriate [42,50]. Specific pathogen-free (SPF) female BALB/c mice (6–8 weeks old) were obtained from a certified laboratory animal center and maintained under standard conditions (22 ± 2 °C, 12 h light/dark cycle) with free access to food and water. All animal experiments were approved by the Experimental Animal Ethics Committee of Heze University (Approval No. 20251216). For the lethal infection model, mice were intraperitoneally injected with the clinical multidrug-resistant *S. aureus* isolate 11413 (2.5 × 10^8^ CFU/200 μL PBS). Each group contained 10 mice. At 2 h post-infection, mice were intraperitoneally treated with ponatinib hydrochloride at doses of 5, 10, or 15 mg/kg once daily for 3 consecutive days. Mouse survival was subsequently monitored and recorded for 7 days. Vancomycin (10 mg/kg) was used as the positive control. For the non-lethal infection model, mice were intraperitoneally injected with 1 × 10^7^ CFU bacterial suspension and treated with ponatinib hydrochloride at 10 mg/kg. Each group contained 5 mice. On day 2 post-infection, mice were sacrificed, and the liver, lungs, and spleen were collected for bacterial burden and inflammatory cytokine analyses. The harvested tissues were weighed, homogenized thoroughly in sterile PBS using a tissue homogenizer, and serially diluted 10-fold. The diluted homogenates were spread onto MHA plates and incubated at 37 °C for 18 h. Colony-forming units (CFUs) were counted and expressed as CFU/g tissue. Meanwhile, the levels of TNF-α, IL-6, and IL-10 in tissue homogenates or serum samples from infected mice were determined using ELISA kits according to the manufacturers’ instructions.

### 4.16. Statistical Analysis

All experiments were performed with at least three biological replicates, and the data are presented as mean ± SD. Statistical analyses were conducted using GraphPad Prism 8.0. Comparisons between two groups were performed using Student’s *t*-test, while comparisons among multiple groups were analyzed using one-way analysis of variance (one-way ANOVA). Mouse survival curves were generated using the Kaplan–Meier method and analyzed by the log-rank test. A value of *p* < 0.05 was considered statistically significant.

## 5. Conclusions

This study is the first to reveal the antibacterial activity of ponatinib hydrochloride against multidrug-resistant *S. aureus* and to elucidate its potential mechanisms of action. Ponatinib hydrochloride not only effectively inhibited MRSA growth and disrupted biofilms but also exhibited a low tendency to induce resistance. Mechanistically, its bactericidal activity was associated with multiple effects, including membrane damage, ROS accumulation, and disruption of energy metabolism. In addition, its pronounced protective efficacy in murine infection models further supports its potential application against drug-resistant *S. aureus* infections. These findings provide new experimental evidence for the repurposing of tyrosine kinase inhibitors in the field of anti-infective therapy and offer new insights into the development of novel anti-infective agents with both antibacterial and host-modulatory properties.

## Figures and Tables

**Table 1:** Bacterial strains used in the study.

Strains	Phenotypic Properties	Source	Ponatinib Hydrochloride (µM)
*S. aureus* ATCC 29213			6.25
*S. aureus* ATCC 43300			12.5
*S. pneumoniae* ATCC 49619			12.5
*E. coli* ATCC25922			>100
*P. aeruginosa* ATCC9027			>100
*S. aureus* 11413	Resistant to PCN, CLI, AZM, TET, VAN and LIN	Pig (Lung)	6.25
*S. aureus* 11527	Resistant to PCN, AZM, TET, VAN and LIN	Pig (Lung)	12.5
*S. aureus* 11502	Resistant to PCN, AZM, TET, VAN and LIN	Pig (Lung)	6.25
*S. aureus* 11633	Resistant to PCN, CLI, AZM, and TET	Pig (Liver)	12.5
*S. aureus* 11427	Resistant to PCN, CLI, AZM, TET and LIN	Pig (Lung)	6.25
*S. aureus* 11243	Resistant to PCN, CLI, AZM, TET and LIN	Pig (Abscess tissue)	25
*S. aureus* 11321	Resistant to PCN, CLI, AZM, TET and LIN	Cattle (Mammary tissue)	12.5
*S. aureus* 11414	Resistant to PCN, CLI, AZM, TET and LIN	Cattle (Mammary tissue)	6.25
*S. aureus* 11523	Resistant to PCN, CLI, AZM, TET and LIN	Cattle (Mammary tissue)	12.5
*S. aureus* 11221	Resistant to PCN, CLI, AZM, TET and LIN	Cattle (Wound tissue)	12.5
*S. aureus* 11332	Resistant to PCN, CLI, TET and LIN	Cattle (Wound tissue)	12.5
*S. aureus* 11334	Resistant to PCN, CLI, AZM, TET and LIN	Cattle (Abscess tissue)	25
*S. aureus* 11276	Resistant to PCN, CLI, AZM, TET and LIN	Cattle (Wound tissue)	12.5
*S. aureus* 11258	Resistant to PCN, CLI, AZM, TET and LIN	Sheep (Abscess tissue)	12.5
*S. aureus* 11406	Resistant to PCN, TET and LIN	Sheep (Abscess tissue)	6.25
*S. aureus* 11418	Resistant to PCN, TET and LIN	Sheep (Abscess tissue)	12.5

PCN, Penicillin; CLI, Clindamycin; AZM, Azithromycin; TET, Tetracycline; VAN, Vancomycin; LIN, Linezolid.

**Table 2:** MIC values of two drugs on *S. aureus* cultured in drug-free medium.

Serial Passaging	MIC (µM)			
	Ponatinib Hydrochloride		Tetracycline	
	ATCC29213	ATCC43300	ATCC29213	ATCC43300
1	6.25	12.5	200	100
2	6.25	12.5	100	200
3	12.5	6.25	100	100
4	12.5	6.25	200	100
5	6.25	12.5	200	200

## Data Availability

The data presented in this study are available on request from the corresponding author.
